# Metabolomics Deciphers Potential Targets of Xuefu Zhuyu Decoction Against Traumatic Brain Injury in Rat

**DOI:** 10.3389/fphar.2020.559618

**Published:** 2020-09-25

**Authors:** Teng Li, En Hu, Pengfei Li, Zhaoyu Yang, Yao Wu, Ruoqi Ding, Xiaofei Zhu, Tao Tang, Yang Wang

**Affiliations:** ^1^Department of Integrated Traditional Chinese and Western Medicine, Institute of Integrative Medicine, Xiangya Hospital, Central South University, Changsha, China; ^2^Department of Respiratory and Critical Care Medicine, The First Affiliated Hospital, Zhengzhou University, Zhengzhou, China

**Keywords:** metabolomics, traumatic brain injury, Xuefu Zhuyu decoction, neuroprotective effects, traditional Chinese medicine

## Abstract

Xuefu Zhuyu decoction (XFZYD) performs multiple functions for traumatic brain injury (TBI) treatment. However, its clinical application is limited by the incomplete exploration of targets and inadequate discussion of mechanisms. We aimed to investigate the metabolic alterations of XFZYD in acute and chronic stages of TBI. Sprague-Dawley rats were randomly divided into the sham, controlled cortical impact (CCI) and XFZYD group. Behavioral and histopathological tests were used to evaluate the neuroprotective effects. Coagulation assays were performed to assess safety. Moreover, we analyzed the metabolomic profiling of hippocampal samples with different time intervals after CCI by high-performance liquid chromatography-tandem mass spectrometry (HPLC-MS/MS). Differential metabolites were screened by multivariate data analysis. To further uncover the association between candidate metabolites and biological interaction networks, we applied bioinformatics analysis using MetaboAnalyst 4.0, STITCH 5.0 and TCMSP. The potential mechanism was verified by ELISA and Western blot. XFZYD ameliorated neurological deficiencies post-CCI without impairing blood coagulation in the rat’s model. Seventeen and fourteen metabolites were filtered on d 3 and 21, respectively. Eleven of potential metabolites were common at these time points, involving two significant pathways (arginine and proline metabolism, phenylalanine, tyrosine and tryptophan biosynthesis). Gamma-aminobutyric acid (GABA) and the related pathways were specifically affected by XFZYD at the acute phase of TBI, while biosynthesis of amino acids was the major pathway influenced at the chronic phase. This study provides broad insights into the therapeutic effects of XFZYD in treating TBI through the whole phases.

## Introduction

Traumatic brain injury (TBI) is a major global health issue (Maas et al., 2017). This disease not only affects individuals and families but also causes a burden to economies at a societal level (Mollayeva et al., 2018). Unfortunately, dozens of clinical trials have failed to improve the outcomes of TBI, mainly resulting from the monotherapies (Kline et al., 2016; Hartings et al., 2019). TBI is heterogeneous and dynamic with various secondary injury mechanisms. Therefore, cocktail strategy acting on multiple targets may enhance the efficacy of TBI treatment (Kline et al., 2016; Somayaji et al., 2018).

Traditional Chinese medicine (TCM) consists of complicated ingredients which meets the criteria of polytherapy (Yang et al., 2016). Hence, TCM represents a promising cocktail strategy for TBI therapy. Xuefu Zhuyu decoction (XFZYD), a traditional Chinese prescription, has proven its merits of treating cardiac-cerebral vascular disease for hundreds of years (Sun et al., 2008; Tao et al., 2019). It is composed of eleven herbs (shown in **Table 1**). XFZYD exhibits a series of pharmacological activities for TBI including ameliorating neurological deficits, reducing inflammation (Xing et al., 2016), improving cognitive impairments (Zhou et al., 2017), etc. XFZYD-derived components, such as amygdalin and neohesperidin, exerted neuroprotective effects through improving neurodegeneration (He et al., 2020) and attenuating apoptosis (Wang et al., 2018), respectively. Despite these researches, the application of XFZYD is limited by its multi-target performances. Conventional molecular methods such as biochemical analyses are difficult to elucidate the comprehensive mechanisms (Wang et al., 2019). To solve the problem, omics methods tend to become a valuable tool to understand the underlying mechanisms (Yang and Lao, 2019).

**Table 1:** Compositions of Xuefu Zhuyu decoction.

Plant name	Latin name	Chinese name	Medicinal part	Ratio	Specimen number
*Prunus persica* (L.) Batsch	*Semen Persicae*	Tao Ren	Seed	8	16080810
*Carthamus tinctorius* L.	*Flos Carthami*	Hong Hua	Flower	6	16121207
*Angelica sinensis* (Oliv.) Diels	*Radix Angelicae Sinensis*	Dang Gui	Root	6	16111801
*Rehmannia glutinosa* (Gaertn.) DC.	*Radix Rehmanniae*	Sheng Di	Root	6	16080402
*Achyranthes bidentata* Blume.	*Radix Achyranthis Bidentatae*	Niu Xi	Root	6	16121909
*Paeonia lactiflora* Pall.	*Radix Paeoniae Rubra*	Chi Shao	Root	4	16071607
*Citrus × aurantium* L.	*Fructus Aurantii*	Zhi Qiao	Fruit	4	16100905
*Glycyrrhiza uralensis* Fisch.	*Radix Glycyrrhizae*	Gan Cao	Root	4	16120303
*Ligusticum striatum* DC.	*Rhizoma Chuanxiong*	Chuan Xiong	Root	3	16120102
*Platycodon grandiflorus* (Jacq.) A. DC.	*Radix Platycodonis*	Jie Geng	Root	3	16102507
*Bupleurum chinense* DC.	*Radix Bupleuri*	Chai Hu	Root	2	16121903

The plant names have been checked with http://www.theplantlist.org.

Metabolomics represents an investigation of metabolites, based on the global metabolic profiles in complex biological matrixes. The holistic view of metabolomics is similar to that of TCM, suggesting that metabolomics has the potential to unveil intricate interactions and multiple factors regulated by XFZYD treatment (Wang et al., 2017). Biofluids are commonly analyzed in metabolomics to reflect systemic metabolic perturbations (Beckonert et al., 2007). Our recent reports of plasma metabolomics indicated that XFZYD exerts neuroprotective effects on the controlled cortical impact (CCI) rats model *via* multiple pathways (Feng et al., 2017; Fu et al., 2019). However, due to the blood-brain barrier, the metabolic alterations in the biofluid may not be in parallel with that in the injured brain (Qian et al., 2019). In this regard, the analysis of brain samples to provide direct information on metabolism is available (Chen et al., 2019).

In the present study, we investigated the hippocampus metabolomics in a rat model of TBI to characterize metabolic changes regulated by XFZYD through the whole phases. Furthermore, we explored the underlying mechanisms *via* network analysis based on metabolomics and bioinformatics. This research provides a better understanding of the neuroprotective effects of XFZYD in treating TBI.

## Materials and Methods

### Preparation of XFZYD

XFZYD consists of 11 herbs: Tao Ren, Hong Hua, Dang Gui, Sheng Di, Niu Xi, Chi Shao, Zhi Qiao, Gan Cao, Chuan Xiong, Jie Geng, and Chai Hu at a dry-weight ratio of 8:6:6:6:6:4:4:4:3:3:2. The herbs were purchased from Hunan Zhenxing Chinese Medicine Co., Ltd (Hunan, China. Drug GMP certificate: HN20150147. Drug Manufacturing Certificate: NO.20150021). They were authenticated by Professor Suiyu Hu, Department of Chinese herbal medicine of Central South University (Changsha, China). Their voucher specimens were deposited at Xiangya Hospital of Central South University. These crude drugs were processed into lyophilized powder (yield=16.9%, *w/w*) as previously described (Wang et al., 2016). Lyophilized powder was dissolved in distilled water to a final concentration of 0.095 g/ml.

### Animals Study

Male specific-pathogen-free Sprague Dawley rats of 7 weeks old, weighing 230 to 250 g, were provided by the Laboratory Animal Centre of Central South University (Changsha, China). They were reared at 25°C in a 12 h dark/light cycle with free access to water and food. All experimental protocols were approved by the Medical Ethics Committee of Central South University and were implemented according to guidelines of Central South University for the care and use of animals.

Rats were randomly divided into three groups: sham, CCI and XFZYD group. CCI was performed as described previously under 3% pentobarbital sodium (60 mg/kg) anesthesia (Xing et al., 2016). The parameters were as follows: impact depth, 5.0 mm; striking speed, 6.0 m/sec; dwell time, 50 msec. The sham-operated rats were subjected to the same anesthesia and craniotomy except for cortical impact. In the XFZYD group, rats were intragastrically administrated with 1.52 g/kg (equivalent to 9 g/kg of raw herbs) XFZYD. The rats in the sham and CCI groups were treated with equal volumes of distilled water.

### Neurological Function Testing

All animals were evaluated using the modified Neurologic Severity Score (mNSS) test, corner turn test, and weight change on the d 3 and 21 after CCI. mNSS is a composite of motor, sensory, reflex abilities and balance tests. It is graded on a scale of 0 to 18, higher scores indicate more serious damage. The corner turn test was repeated 10 trials for each rat and the times of right turns were calculated. The normal rats turned about half of the time towards the right. Therefore, the higher the time, the worse the neurological deficits. The rats were weighed before and after the injury, and the percentage of weight change was calculated. The details of these tests were provided in our previous work (Xing et al., 2016; Li et al., 2019).

### Sample Collection and Pretreatment

All rats were sacrificed under intraperitoneal injection of 3% pentobarbital sodium (60 mg/kg) anesthesia on d 3 and 21. Blood samples for coagulation tests were collected into vacuum tubes (blue cap) containing sodium citrate in a 9:1 volume ratio. For LC-MS analysis of herbal ingredients, the samples were collected into vacuum tubes (green cap) containing heparin sodium. All tubes were mixed by inverting the tubes 8 to 10 times immediately after the blood draw and centrifuged at 3,000 rpm (10 min, 4°C) to obtain plasma. The plasma samples were stored at −80°C before being detected.

The rats for hematoxylin-eosin (HE) staining were perfused transcardially with ice-cold saline, followed by 4% paraformaldehyde. The brains were removed and fixed in 4% paraformaldehyde for 6 h, then changed into phosphate-buffered saline (PBS, 0.01 mol/L, pH=7.4), embedded in paraffin.

The rats for HPLC-MS/MS were perfused transcardially with ice-cold saline. Hippocampus is one of the most vulnerable brain areas post-TBI. Hippocampal injury deems to reflect the aggregated effects of the trauma-induced cellular loss that develops over time (Yan et al., 2016). Therefore, the hippocampal tissues surrounding the injured region were obtained and preserved at −80°C.

### Qualitative Analysis of XFZYD

The standard reference materials of amygdalin, narirutin, neohesperidin and digoxin were purchased from Yuanye Bio-Technology Co., Ltd (Shanghai, China). Digoxin is not the endogenous compound of XFZYD and plasma, and it does not obviously interfere with the retention times of all three analytes. Qualitative analysis was carried out using an LC-MS system (Shimadzu 8050, Kyoto, Japan) in negative ion mode. The plasma samples were added with digoxin, and then vortex mixed for 1 min and centrifuged for 15 min (13,000 rpm, 4°C) after being added with acetonitrile. The obtained supernatants were dried in a nitrogen dryer, diluted with 10% acetonitrile-water, repeated the extraction above, and injected into the LC-MS for analysis.

### Histopathology

Brain sections (each 5-μm) were deparaffinized in xylene, hydrated by ethanol, stained with HE reagent (Beijing Solarbio Science & Technology Co., Ltd, Beijing, China). The morphology of hippocampal neurons was observed under a light microscope.

### Coagulation Assays

Prothrombin time (PT), activated partial thromboplastin time (APTT), thrombin time (TT) and fibrinogen content (FIB) were examined following the manufacturer’s instructions by a coagulometer (RAC-030 automatic coagulation analyzer, Rayto Life and Analytical Sciences Co., Ltd, Shenzhen, China).

### Chemicals and Reagents

Ammonium acetate (NH_4_OAc), ammonium hydroxide (NH_4_OH) and MeOH were purchased from Sigma-Aldrich (St. Louis, MO, USA), Acetonitrile (ACN) and H_2_O were purchased from J.T.Baker (PA, USA). All the remaining reagents were of analytical grade.

### High-Performance Liquid Chromatography-Tandem Mass Spectrometry (HPLC-MS/MS) Analysis

Each tissue sample was weighed and extracted on ice with MeOH: ACN (1:1, v/v). Followed by 30 s of vortex mixing and 10 min of sonicating after being added with methionine sulfone (0.2 mmol/L). The obtained supernatants were dried in a nitrogen dryer. The dried sample was reconstituted in 40 µl/mg.pro of ACN: H_2_O (1:1, v/v), swirled for 30 s and sonicated for 10 min, then centrifuged for 15 min (20000 rpm, 4°C) to obtain supernatants. The supernatants were transferred to HPLC vials for further analysis. The quality control (QC) samples were prepared by mixing 10 µl of every tissue sample as same as the experiment samples.

1,260 infinity high-performance liquid chromatography (Agilent, CA, USA) was used for liquid chromatography separation. Samples were separated on an amide column (Waters, MA, USA). Mobile phase A consisted of water mixed with 25 mmol/L NH_4_OAc and 25 mmol/L NH_4_OH. Mobile phase B contained ACN. The injection volume was 4 µl. The flow rate was 0.4 ml/min. MS analysis was performed on the Q-Exactive MS/MS (Thermo, MA, USA) in both positive and negative ion modes. The ion spray voltage was maintained at 3.5 kV with scanning range from 60 to 900 *m/z*. The aux gas heater temperature was 400°C. The capillary temperature was set at 350°C.

### Metabolic Profiling and Pathway Analysis

Raw files from HPLC-MS/MS were submitted to Compound Discover 2.1 (Thermo, MA, USA), and processed with untargeted metabolomics workflow: retention time alignment, unknown compound detection, elemental compositions prediction, gaps filling and chemical background hiding, normalization, etc. The relative standard deviation (RSD) of differential metabolites intensities in the QC samples was calculated. Metabolites with an RSD of less than 30% were reserved for statistical analysis. The mean-centered and pareto-scaled data were analyzed by principal component analysis (PCA), partial least-squares discriminant analysis (PLS‐DA) modeling and permutation test using ropls R package in R software (version 3.6.0). Validation of the mathematical model was performed by 7-round cross-validation and followed by a permutation test with 200 iterations. Variable importance in the projection (VIP) was applied to choose potential biomarkers. The accurate mass and MS/MS information of metabolites were further identified by mzCloud (https://www.mzcloud.org/) and ChemSpider (https://www.chemspider.com/) with four databases selected (BioCyc, HMDB, KEGG, LipidMAPS).

The identified metabolites were assessed with the Shapiro-Wilk test for normality. Next, Mann-Whitney U-test was performed for nonparametric data and two-tailed Student’s t-test was performed for parametric data between two groups by SPSS 23.0 (International Business Machines Corp., Armonk, NY, USA). Significant differential expressions were those with an adjusted *P*-value [i.e., false discovery rate (FDR)] of <0.05. The signiﬁcantly differential metabolites were selected by FDR<0.05 and VIP>1. The multivariate receiver operating characteristic (ROC) curves and predictive plots were applied to validate the accuracy of the differential metabolites. Upset plot and heat maps were displayed using the pheatmap package and the UpSetR package in R, respectively. To explore the molecular mechanism, the altered metabolites were imported into MetaboAnalyst 4.0 (https://www.metaboanalyst.ca/) and Search Tool for Interacting Chemicals (STITCH 5.0, http://stitch.embl.de/) by searching “Rattus norvegicus,” and plotted with ggplot2 R package and Cytoscape 3.7.2 (the Cytoscape Consortium, CA, USA).

### Network Pharmacology Analysis

To explain the relation among active compounds, proteins and metabolites, network construction was performed by Cytoscape 3.7.2. The metabolite-related targets were selected from STITCH. These targets were imported into the UniProt database (http://www.uniprot.org/) to standardize the protein names. The compounds of XFZYD and their biological targets were collected from the Traditional Chinese Medicine Systems Pharmacology Database and Analysis Platform (TCMSP, http://tcmspw.com/tcmsp.php). TCMSP is a systems pharmacology platform of Chinese herbal medicines that depicts the relationships among drugs, targets, and diseases (Ru et al., 2014). Compounds were filtered based on oral bioavailability (OB) ≥30% and drug-likeness (DL) ≥0.18. The compound-related targets were also filtered out from TCMSP.

### Enzyme-Linked Immunosorbent Assay (ELISA)

The contents of l-arginine in the hippocampus were determined with commercial kits (specific for rats, Shanghai Zhuocai Biotechnology Co., Ltd., China) according to the manufacturer’s protocols. The optical densities were detected at 450 nm by a microplate reader (HEALES MB-530, Shenzhen Huisong Technology Development Co., Ltd, China). The concentration of l-arginine was calculated with the standards curves.

### Western Blot

Hippocampal tissues were washed by ice-cold PBS and lysed in RIPA buffer, followed by homogenizing mechanically. The homogenates were centrifuged at 12000 rpm for 15 min at 4°C. After electrophoresis on SDS-PAGE gels, proteins were transferred to membranes and blocked with 5% non-fat milk. Then, the blots were incubated with rabbit-anti-rat arginase-1 (ARG1) antibody (93668S, 1:1000; CST, USA), neuronal nitric oxide synthase (NOS1) antibody (ab76067, 1:1000; Abcam, UK), inducible nitric oxide synthase (NOS2) antibody (ABP51974, 1:1000; Abbkine, China), endothelial nitric oxide synthase (NOS3) antibody (#32027, 1:1000; CST, USA) and mouse-anti-rat β-actin antibody (66009-1-Ig, 1:5000; Proteintech, USA) at room temperature. After washing with PBST for three times, the blots were incubated with HRP goat-anti-rabbit IgG (SA00001-2, 1:6000; Proteintech, USA) and HRP goat-anti-mouse IgG (SA00001-1, 1:5000; Proteintech, USA) for 90 min. The color reaction was performed by ECL reagents. The band density was visualized after exposure to x-ray film and analyzed using the quantity one software (Bio-Rad, USA).

### Statistical Analysis

The results of neurological function tests and coagulation assays were expressed as mean ± SD. The differences were compared by one-way analysis of variance (ANOVA) with Fisher’s LSD test. *P*<0.05 was considered statistically significant using SPSS 26.0.

## Results

### Quality Control of XFZYD

**Figure 1:**
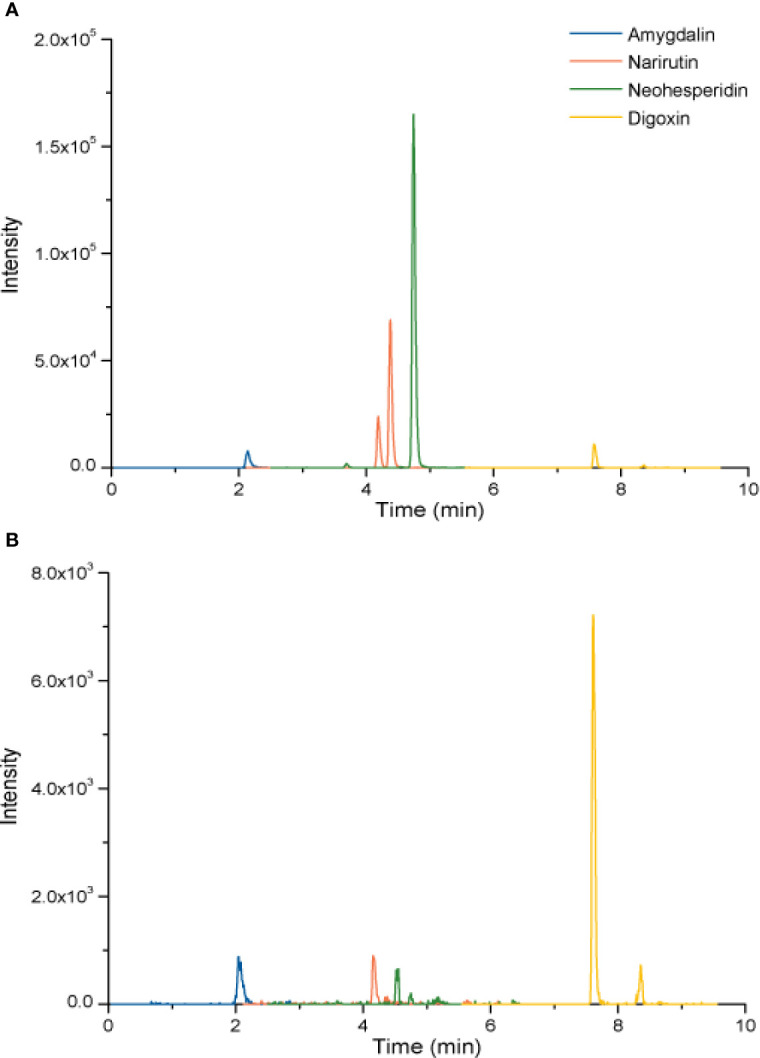
LC chromatogram of amygdalin, narirutin, and neohesperidin of standard agents **(A)** and XFZYD **(B)** in plasma samples post-CCI.

To investigate the quality control of XFZYD, we applied the LC-MS method. Amygdalin is the hub compound of Tao Ren. Narirutin and neohesperidin are the main constituents of Zhi Qiao. Digoxin was the internal reference. As shown in **Figure 1**, amygdalin, narirutin, neohesperidin and digoxin were identified with each retention time of 2.08 ± 0.040 min, 4.18 ± 0.016 min, 4.53 ± 0.006 min and 7.61 ± 0.004 min, respectively. The coefficients of variation of the four components were less than 2%, suggesting the stability of the method.

### XFZYD Exerts Neuroprotective Effects on CCI Rats

**Figure 2:**
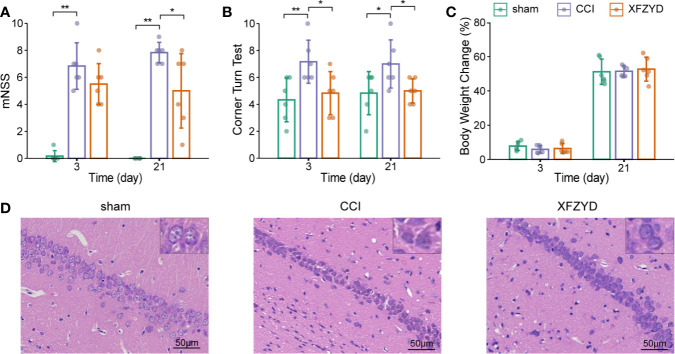
Effects of XFZYD on neurological status after CCI. The mNSS **(A)**, corner turn test **(B)**, body weight change **(C)** plots of sham and CCI rats administrated with either distilled water or XFZYD on d 3 and 21. Representative images of HE-stained (×400) hippocampus in the CA1 region on d 21 **(D)**. Data are presented as mean ± SD, n = 6 rats per group, one-way ANOVA followed by Fisher’s LSD test. *P<0.05, **P<0.01.

We assessed mNSS, corner turn test and body weight growth to investigate whether XFZYD influences neurological recovery. On d 3 and 21, the higher mNSS (**Figure 2A**) and increased right turn times (**Figure 2B**) were observed in the CCI group compared with the sham group (*P*<0.05). These results proved the successful models we constructed. On d 3, compared with CCI rats, the XFZYD group showed no significant differences for mNSS, but there was a trend towards lower levels (**Figure 2A**). However, XFZYD notably declined the scores of the corner turn test (**Figure 2B**). On d 21, XFZYD induced a remarkable decrease in mNSS and corner turn test compared with CCI rats (**Figures 2A, B**). Additionally, the three groups showed comparable weight gains throughout 21 d, irrespective of treatment (**Figure 2C**).

HE staining was used to observe neurons in the cornu ammonis 1 (CA1) region of the hippocampus. Cells in the sham group were arranged regularly, clear and intact in structure (**Figure 2D**). CCI resulted in karyopyknosis and neural reduction on d 21. The damage was prominently reduced and cells were aligned properly after XFZYD administration. These results indicated that XFZYD treatment ameliorated neurological deficits in CCI rats.

### XFZYD Causes No Bleeding Risk After CCI

**Figure 3:**
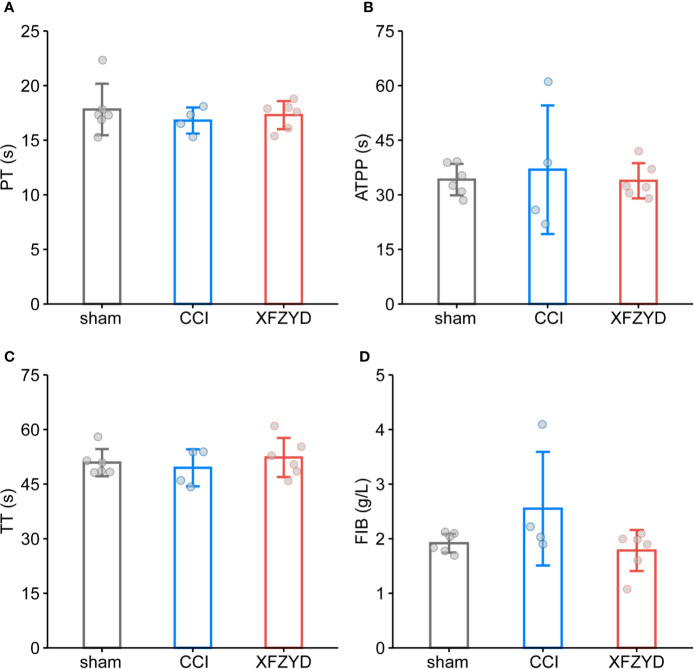
Effect of XFZYD on coagulation indexes after CCI. PT **(A)**, APTT **(B)**, TT **(C)**, FIB **(D)** plots of sham and CCI rats administrated with either distilled water or XFZYD on d 3. Data are expressed as mean ± SD, n = 4 to 6 rats per group, one-way ANOVA followed by Fisher’s LSD test.

Given that XFZYD may remove blood stasis by activating blood circulation (Han et al., 2016; Zhao et al., 2019), we tested whether XFZYD influences the coagulation parameters (PT, APTT, TT, FIB). The measurements showed no significant difference among these groups on d 3 (**Figure 3**), which demonstrated that XFZYD is safe for treating TBI in the acute phase.

### XFZYD Regulates Metabolic Profiles in CCI Rats

**Figure 4:**
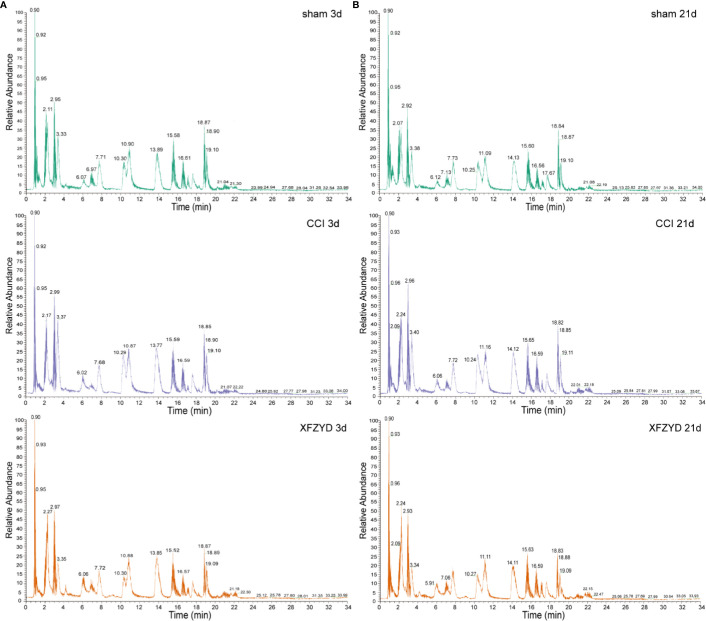
Typical total ion chromatogram (TIC) of rat hippocampal samples on d 3 **(A)** and d 21 **(B)** in the positive ion mode.

To define metabolomic alterations induced by XFZYD that contributes to neural protection, we performed HPLC-MS/MS of hippocampal samples dissected from all groups. As shown in **Figures 4A, B**, we found differences in the levels of major metabolites among the three groups on d 3 and 21.

**Figure 5:**
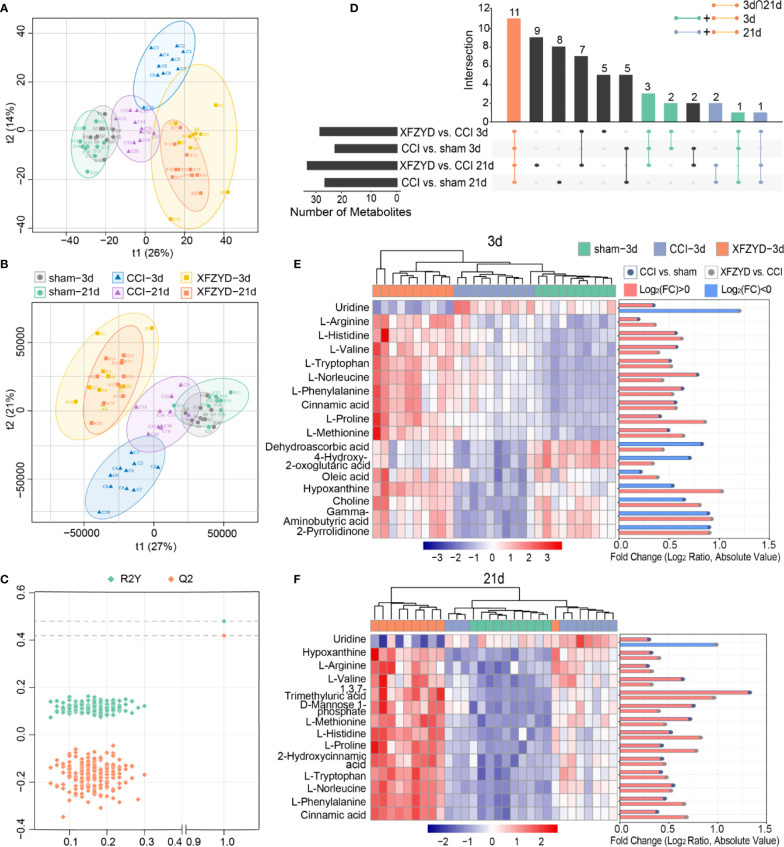
Multivariate statistical analysis of the sham, CCI, and XFZYD groups on d 3, 21. **(A)** PCA score plot; **(B)** PLS-DA score plot; **(C)** cross-validation plot of PLS-DA model; **(D)** Upset plot depicted the intersections of metabolites in diverse combinations. Colored dots with connecting lines denote rows included in the intersection. Heat map and log-fold change visualization of the relative levels of remarkable metabolites on d 3 **(E)** and d 21 **(F)**. Data were conducted based on the average intensity ratio and the Pearson correlation method. The degree of change is shown by color depth. The higher content is marked in red, the lower level is marked in blue.

To further identify the metabolic classification and movement from d 3 to 21 post-operation, PCA (**Figure 5A**) and PLS-DA (**Figure 5B**) were applied by R script. In the unsupervised PCA score plot, the R2X was 0.556 calculated by two principal components (t1 and t2). In the PLS-DA score plot, the R2X, R2Y and Q2 parameters were 0.558, 0.48 and 0.419, respectively. The validation plot (**Figure 5C**) supported the reliability of the PLS-DA model. We observed distinct separations between sham and CCI groups according to PCA and PLS-DA patterns, reflecting the successful modeling process. Samples of CCI and XFZYD rats were well distinguished, indicating that XFZYD caused obvious endogenous metabolite changes post-CCI. Furthermore, there were time-dependent changes in response to CCI.

Next, we examined the metabolic alterations which changed in disease progression. With the standard of FDR<0.05 and VIP>1, we selected seventeen and fourteen metabolites among the variables on d 3 and 21 (**Table 2**), respectively. Eleven of them were common at two time points (**Figure 5D**). The MS/MS spectra of differential metabolites were showed in **Figure S1**. The significance and reliability of the identified metabolites were verified by ROC curve analysis and predictive accuracy plots (**Figure S2**). The area under the ROC curve (AUC) is an indicator of diagnostic efficiency. The AUC values for all metabolites were greater than 0.95, indicating that these metabolites may denote the significant metabolites.

**Table 2:** Basic information on significant metabolites in different groups.

Metabolites	Formula	KEGG	HMDB	CCI vs. sham (3d)		XFZYD vs. CCI (3 d)		CCI vs. sham (21 d)		XFZYD vs. CCI (21 d)	
				VIP	FDR	VIP	FDR	VIP	FDR	VIP	FDR
Hypoxanthine	C_5_H_4_N_4_O	C00262	0000157	7.23	0.000	12.95	0.000	9.27	0.012	10.94	0.004
Uridine	C_9_H_12_N_2_O_6_	C00299	0000296	3.40	0.007	6.47	0.000	3.65	0.034	6.29	0.000
l-Phenylalanine	C_9_H_11_NO_2_	C00079	0000159	3.28	0.000	3.64	0.009	3.80	0.006	5.82	0.000
l-Arginine	C_6_H_14_N_4_O_2_	C00062	/	1.69	0.045	2.87	0.000	2.81	0.028	3.34	0.008
Cinnamic acid	C_9_H_8_O_2_	C10438	0000567	2.22	0.000	2.75	0.009	2.52	0.009	4.47	0.000
l-Norleucine	C_6_H_13_NO_2_	C01933	0001645	3.06	0.000	2.66	0.011	3.44	0.002	3.95	0.000
l-Proline	C_5_H_9_NO_2_	C00148	0000162	1.26	0.000	2.41	0.003	1.94	0.005	3.12	0.000
l-Histidine	C_6_H_9_N_3_O_2_	C00135	0000177	1.42	0.003	1.80	0.000	1.58	0.004	2.84	0.000
l-Methionine	C_5_H_11_NO_2_S	C00073	0000696	1.02	0.007	1.63	0.000	2.21	0.000	1.86	0.000
l-Valine	C_5_H_11_NO_2_	C00183	0000883	1.28	0.000	1.18	0.024	1.86	0.000	1.21	0.039
l-Tryptophan	C_11_H_12_N_2_O_2_	C00078	0013609	1.11	0.000	1.57	0.000	1.55	0.011	1.94	0.000
Choline	C_5_H_13_NO	C00114	0000097	5.72	0.000	7.18	0.000	/	/	/	/
Gamma-Aminobutyric acid	C_4_H_9_NO_2_	C00334	00112	4.76	0.000	5.03	0.000	/	/	/	/
Dehydroascorb-ic acid	C_6_H_6_O_6_	C00425	0001264	4.28	0.000	2.57	0.011	/	/	/	/
2-Pyrrolidinone	C_4_H_7_NO	C11118	0002039	1.83	0.000	1.86	0.000	/	/	/	/
4-Hydroxy-2-oxoglutaric acid	C_5_H_6_O_6_	C01127	0002070	3.10	0.000	1.71	0.024	/	/	/	/
Oleic acid	C_18_H_34_O_2_	C00712	0000207	1.71	0.046	3.58	0.005	/	/	/	/
1,3,7-Trimethyluric acid	C_8_H_10_N_4_O_3_	C16361	0002123	/	/	/	/	1.34	0.000	1.43	0.000
2-Hydroxycinna-mic acid	C_9_H_8_O_3_	C01772	0002641	/	/	/	/	1.41	0.008	1.62	0.004
alpha-d-Mannose 1-phosphate	C_6_H_13_O_9_P	C00636	/	/	/	/	/	2.63	0.000	1.98	0.004

Subsequently, heat maps were adopted to visualize their relative concentrations in each individual (**Figures 5E, F**). On d 3, eight metabolites [uridine, dehydroascorbic acid, 4-hydroxy-2-oxoglutaric acid, oleic acid, hypoxanthine, choline, gamma-aminobutyric acid (GABA) and 2-pyrrolidinone] were reversed after XFZYD administration compared with the CCI group. The amounts of the rest nine metabolites (l-arginine, l-histidine, l-valine, l-tryptophan, l-norleucine, l-phenylalanine, cinnamic acid, l-proline, and l-methionine) were sustained at higher levels compared with the CCI samples. On d 21, uridine was reversed by XFZYD compared with the CCI rats, while thirteen metabolites (hypoxanthine, l-arginine, l-valine, 1,3,7-trimethyluric acid, d-mannose 1-phosphate, l-methionine, l-histidine, l-proline, 2-hydroxycinnamic acid, l-tryptophan, l-norleucine, l-phenylalanine, and cinnamic acid) were up-regulated continuously. All common metabolites showed a similar trend in the XFZYD group compared with the CCI group on d 3 and 21. These data reveals that XFZYD exerts therapeutic effects on CCI rats through multiple targets.

### Metabolic Pathway and Integrated Network Analysis

**Figure 6:**
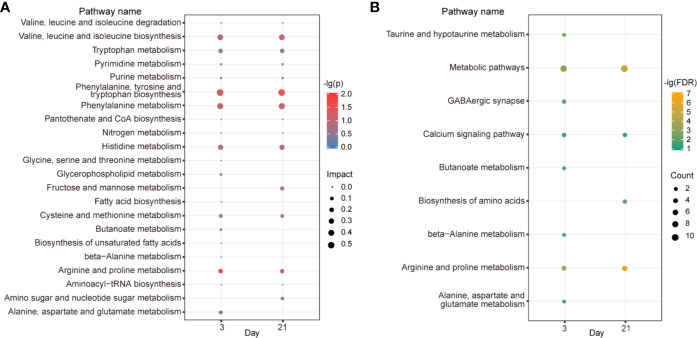
Metabolic pathway and correlation network analysis. KEGG pathway enrichment by MetaboAnalyst **(A)**. The color depth and bubble size indicate -lg(*P*) values and impact of the pathway, respectively. Pathway analyses by STITCH **(B)**. The color depth shows the -lg(FDR) values. Bubble size depicts the numbers of proteins identified in the pathway.

To uncover the functional association between the candidate metabolites and the biological interaction networks, we conducted a bioinformatics analysis using MetaboAnalyst 4.0. As shown in **Figure 6A**, there were considerable overlaps of pathways between the two time points, suggesting common mechanisms included in the two phases. The criterion of the potential targeted pathway was set at *P*<0.05 and impact>0.1 in MetaboAnalyst computation. Phenylalanine, tyrosine and tryptophan biosynthesis, as well as arginine and proline metabolism, were screened out for d 3. Phenylalanine, tyrosine and tryptophan biosynthesis was selected for d 21. To collect more information about the metabolites, STITCH 5.0 was used to predict the proteins and pathways involved in metabolic profiles. As shown in **Figure 6B**, on d 3, there were eight pathways impacted: arginine and proline metabolism, taurine and hypotaurine metabolism, GABAergic synapse, butanoate metabolism, beta-alanine metabolism, alanine, aspartate and glutamate metabolism, calcium signaling pathway. On d 21, there were three pathways affected significantly: arginine and proline metabolism, biosynthesis of amino acids and calcium signaling pathway.

**Figure 7:**
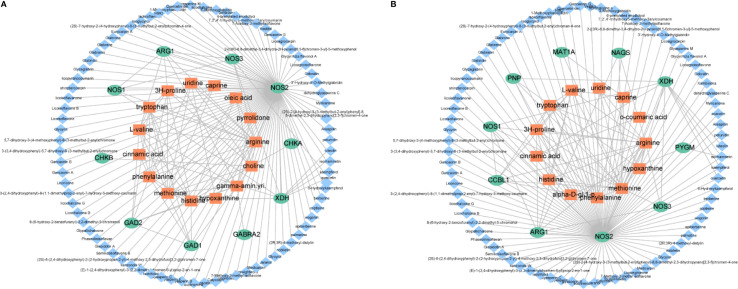
Illustration of the compound-protein-metabolite networks on d 3 **(A)** and d 21 **(B)**. Diamond-shaped blue nodes represent the active compounds. Rounded green nodes represent the proteins. Quadrangular orange nodes represent the metabolites.

To visualize the relationships among the potential metabolites, targets and ingredients of XFZYD, we constructed a compound-target-metabolite network. As shown in **Figure 7**, seventy-nine compounds regulated ten targets and fifteen potential metabolites on d 3, seventy-nine compounds interacted with ten targets and thirteen potential metabolites on d 21. These results reveal that NOS1, NOS2, NOS3 and ARG1 may play key roles in the XFZYD treatment on TBI. These targets belong to arginine and proline metabolism with l-arginine as the key metabolite.

### Verification of the Mechanism of XFZYD in Treating CCI Rats

**Figure 8:**
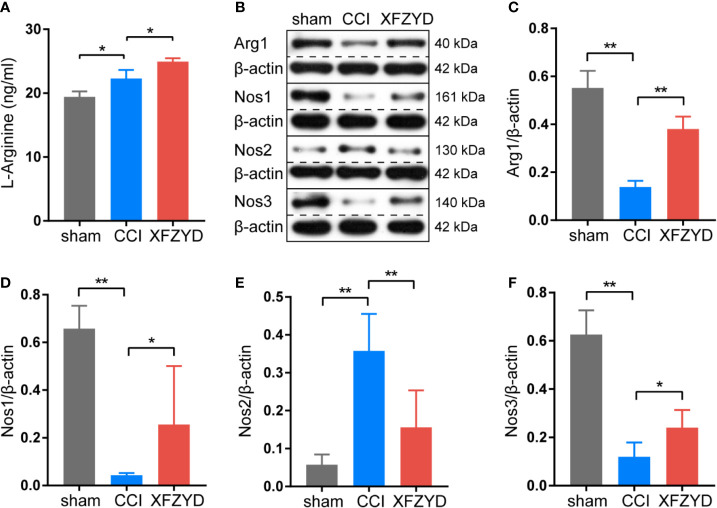
Effects of XFZYD on the expression levels of l-arginine, ARG1, NOS1, NOS2, NOS3 in CCI-induced rats. **(A)** The content of l-arginine among sham, CCI and XFZYD groups on d 21 post-trauma. Data are presented as mean ± SD, n = 3 rats per group, one-way ANOVA followed by Fisher’s LSD test. *P < 0.05, **P < 0.01. Representative western blots of ARG1, NOS1, NOS2 and NOS3 **(B)** and their quantiﬁcations **(C–F)** among sham, CCI and XFZYD groups on d 21 post-trauma. Data are presented as mean ± SD, n = 5 rats per group, one-way ANOVA followed by Fisher’s LSD test. *P < 0.05, **P < 0.01.

Combining the network analysis with the KEGG pathway exploration, we speculated that arginine and proline metabolism may act as a significant pathway during the process of XFZYD in treating TBI. We examined the expression of l-arginine in the hippocampus by ELISA (**Figure 8A**). Compared with the sham group, l-arginine increased significantly after CCI. After XFZYD treatment, l-arginine increased continuously compared with the CCI group. These results are consistent with the examination of metabolomics. Western blot analyses showed that ARG1, NOS1 and NOS3 were notedly down-regulated, while NOS2 was markedly up-regulated after CCI, and XFZYD reversed their expressions significantly (**Figures 8B–F**).

## Discussion

**Figure 9:**
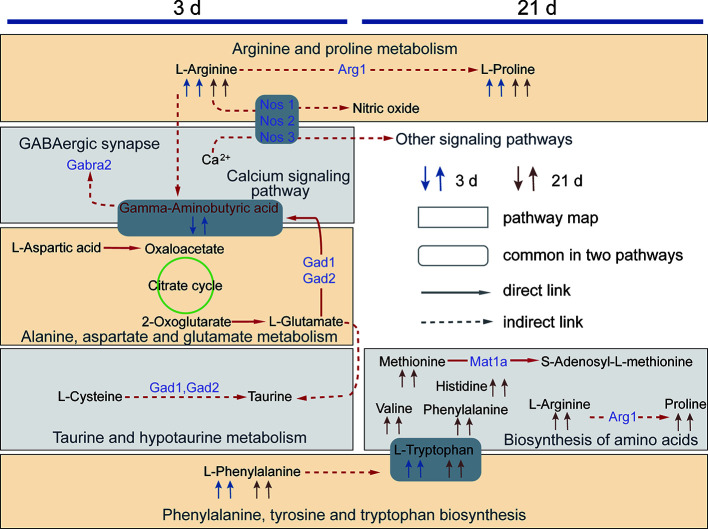
The network diagram of the metabolic pathways regulated by XFZYD on d 3 and 21. The first and the second arrow in 3 d and 21 d denotes metabolite changes in CCI vs. sham and XFZYD vs. CCI, respectively. A square frame represents a metabolic pathway, a round rectangle frame represents biochemical factors that are common in two pathways. A solid arrow indicates molecular interaction between two chemicals, a dotted arrow indicates an indirect or unknown reaction. Purple words denote proteins predicted by STITCH.

This study revealed the effects of XFZYD on the neurometabolic states of phase-specific CCI rats. Our data showed that there are eleven potential metabolites shared in both acute and chronic stages. Four of them involving in the regulations of two common pathways. XFZYD affects differential metabolites and pathways at each time point. These results highlight deep insights into multiple regulations of XFZYD in treating TBI through the whole phases. The comprehensive metabolic networks were mapped based on the relationships between biochemical factors and differential metabolites (**Figure 9**).

In our previous study, we have found the classic dosage of XFZYD (9 g/kg in rat), as defined by Qingren Wang in the document “Yi Lin Gai Cuo,” delivers the optimal effects (Xing et al., 2016). In addition, the experimental investigation indicated that 9 g/kg of XFZYD facilitates neurological recovery post-CCI (Feng et al., 2017). In this study, our behavioral measures support the above conclusions. Furthermore, HE staining showed that XFZYD alleviates the neuronal damage of the hippocampus. Based on the TCM theory, the treatment principle of XFZYD is to promote blood circulation and remove blood stasis (Tao et al., 2019). This “blood-activating” property raises concerns about the possible anticoagulation effects (Fung et al., 2017), which may increase the bleeding risk during the acute phase of TBI. Our study confirmed that XFZYD induces no impairments of clotting functions for CCI rats. Hence, this decoction tends to be safe to treat TBI.

The metabolic alterations caused by XFZYD treatment is a dynamic process, which affecting amino acids (GABA, l-tryptophan, l-phenylalanine, l-arginine, l-proline, l-histidine, l-methionine, and l-valine), nucleotides (uridine and hypoxanthine), vitamins (choline and dehydroascorbic acid), lipid (oleic acid) and others (such as 2-pyrrolidinone, 4-hydroxy-2-oxoglutaric acid and alpha-d-mannose 1-phosphate). Most of the deteriorations in amino acids occur at early-phases post-TBI (6–24 h), which may affect various molecular pathways. With the passage of time, the system tries to maintain homeostasis (Amorini et al., 2017). Similar to our study, higher levels of amino acids have also been found in the brain at the acute phase from an ischemic stroke rat model (Wesley et al., 2019). In this study, all identified amino acids are increased in CCI vs. sham group and XFZYD vs. CCI group on d 3 and 21.

Arginine and proline metabolism, as well as phenylalanine, tyrosine and tryptophan biosynthesis, are predominantly impacted. l-arginine serves as a substrate to synthesize nitric oxide (NO) by NOS (Madan et al., 2018). ARG1 competes with NOS for l-arginine to indirectly generate proline, which is required for extracellular matrix remodeling (Li and Wu, 2018). NO released from NOS1 and NOS3 vasodilates intracranial vessels and increases cerebral blood flow (Costa et al., 2016; Rami and Robert, 2018) . NOS2 is the biomarker of pro-inflammatory microglia (M1), and ARG1 is the biomarker of anti-inflammatory microglia (M2). M1 and M2 play important regulatory roles in neuroinflammation (Zhou et al., 2020). In the CCI group, the contents of NOS1 and NOS3 are reduced, indicating the vascular dysfunction caused by CCI. The contents of NOS2 are increased after CCI, implicating the inflammation happens in the hippocampus. l-arginine is upregulated to supply the substrate needed for the reaction of NOS2. XFZYD significantly elevates the expressions of NOS1 and NOS3, by which to regulate the vascular homeostasis. XFZYD also improves the M1/M2 phenotype polarization of microglia accompanied with lower NOS2 and higher ARG1, by which to alleviate the neuroinflammation of CCI rats. Increased l-arginine in the XFZYD group is provided for the activities of NOS1, NOS3 and ARG1.

For the phenylalanine, tyrosine and tryptophan biosynthesis, CCI and XFZYD groups elevate the levels of l-phenylalanine and l-tryptophan, which belong to aromatic amino acids. In addition to becoming components for protein synthesis and providing higher trophic levels (Fernstrom and Fernstrom, 2007), tryptophan and phenylalanine are also the obligatory substrates for synthesizing the neurotransmitter serotonin and dopamine, respectively (Durham et al., 2017). Serotonin and dopamine improve memory, mood and cognitive functions of chronic TBI patients (Leiser et al., 2015; Lan et al., 2019). Additionally, l-histidine, l-methionine, and l-valine are the essential amino acids needed in humans for growth and brain repair. An accumulation of these amino acids may indicate changed neurotransmitter levels and protein generation in the brain due to injury (Dauncey, 2014; Yoshikawa et al., 2014). In this study, CCI rats showed normalization of the beneficial amino acids after 3 days from trauma, indicating the reparative process acts in the injured brain. Moreover, XFZYD accelerates the recovery process.

Hypoxanthine and uridine are also screened as potent metabolites shared on d 3 and 21. At the acute stage, xanthine oxidase exacerbates brain injury by generating free radicals through converting hypoxanthine to xanthine and xanthine to uric acid (Mink and Johnston, 2007). Consistent with the previous result (Zheng et al., 2019), hypoxanthine is decreased on d 3 post-CCI and reversed by XFZYD. We speculate that XFZYD reduces brain damage by preventing hypoxanthine degradation, further inhibiting the production of oxygen-free radicals. Additionally, hypoxanthine plays a significant role in the catabolism of ATP, which leads to an improved energy state in the brain (Zhang et al., 2017). Others have also shown that elevation of hypoxanthine exerts a neuroprotective effect in the impaired brain (Shen et al., 2005; Brown et al., 2017). Therefore, during the later reparative phase, hypoxanthine is up-regulated in CCI and XFZYD groups. XFZYD leads to a lower content of uridine than the CCI group. TBI destabilizes cellular membranes, which creates a need for sustained biosynthesis of neuronal membrane phospholipids (Thau-Zuchman et al., 2019). Uridine serves as a substrate synthesizing phospholipids. We hypothesize that XFZYD exerts beneficial effects by regulating the conversion of uridine to phospholipids in TBI.

Although lots of metabolites levels display similar changes during the acute and chronic stages, the variation patterns of some metabolites are clearly different. There are metabolites responding specifically to the XFZYD treatment during the acute phase of TBI. GABA is the principal inhibitory neurotransmitter in the brain, which is in concert with glutamate to control the balance of excitation and inhibition in many brain circuits (Luo et al., 2019). Following TBI, the amount of GABA in neuronal synapses decreases, but the release of glutamate elevates promptly. The disruption of the balance results in the aggravation of lesion (Guerriero et al., 2015). Coherent with the previous literature (Luo et al., 2019), GABA is down-regulated in the hippocampus post-trauma. XFZYD reverses GABA content to normal, leading to the attenuation of glutamate and the improvement of micro-environment in injured sites. XFZYD modulates the contents of GABA mainly through GABAergic synapse and alaine, aspartate and glutamate metabolism. 2-pyrrolidinone is a lactam cyclization product of GABA. The elevated level of 2-pyrrolidinone in XFZYD rats is in accord with the higher level of GABA compared with the CCI group. In the present research, we firstly reported the detection of 2-pyrrolidinone in the hippocampus of CCI rats following XFZYD treatment. In addition, choline is essential for the synthesis of structural cell membrane phospholipids and the neurotransmitter acetylcholine (Guseva et al., 2008). Consistent with the previous works (Casey et al., 2008), choline is lower after CCI. This abnormality is restored by XFZYD. Oxidative stress is a critical secondary process resulting from TBI. Free radicals attack membrane lipids, initiate the chain reaction of lipid peroxidation and lead to tissue damage (Kline et al., 2016). Dehydroascorbic acid is a blood-brain barrier transportable form of ascorbic acid. Previous work has proven that it mediates a prominent neuroprotective effect in experimental stroke (Huang et al., 2001). Our study showed that XFZYD up-regulates the content of dehydroascorbic acid, which may be converted into ascorbic acid and abrogated oxidative stress in TBI (Covarrubias-Pinto et al., 2015). To the best of our knowledge, this is the first time to confirm the alteration of dehydroascorbic acid in the hippocampus of TBI. 1, 3, 7-trimethyluric acid responds specifically to XFZYD treatment during the chronic phase of TBI. This metabolite is a methyl derivative of uric acid, which functions as an antioxidant and free radical scavenger to prevent lipid peroxidation (Nishida, 1991). We believe that XFZYD alleviates oxidative stress *via* increasing 1, 3, 7-trimethyluric acid. These circumstances explained the time-dependent metabolic differences of XFZYD in treating TBI.

## Conclusion

Taken together, this metabolomics work highlights an overview of multiple functions regulated by XFZYD for TBI treatment. Furthermore, XFZYD evokes metabolic responses on the hippocampus through the whole phases of TBI without bleeding risk. These observations provide us with broad insight into the acute and chronic therapeutic effects of XFZYD on TBI.

## Data Availability Statement

The raw data supporting the conclusions of this article will be made available by the authors, without undue reservation.

## Ethics Statement

The animal study was reviewed and approved by Medical Ethics Committee of Central South University.

## Author Contributions

YWa, TL, and TT conceived and designed the experiments. TL, PL, ZY, YWu, RD, and XZ performed the experiments. TL and EH analyzed the data and visualized the figures. TL drafted the manuscript. YWa and TT revised it. All authors contributed to the article and approved the submitted version.

## Funding

This work was supported by the National Natural Science Foundation of China (No. 81673719 and No. 81973665), Hunan Provincial Natural Science Foundation of China (No. 2019JJ30042) and Innovation-Driven Project of Central South University (No. 2020CX047).

## Conflict of Interest

The authors declare that the research was conducted in the absence of any commercial or financial relationships that could be construed as a potential conflict of interest.
