# Supplementary material for: Metabolomics Deciphers Potential Targets of Xuefu Zhuyu Decoction Against Traumatic Brain Injury in Rat
Source: Front Pharmacol. 2020 Sep 25;11:559618. doi: 10.3389/fphar.2020.559618 (PMC7546399; doi:10.3389/fphar.2020.559618)
Supplement: Supplementary file 1 [file Table_1.docx]

**Table 1** Compositions of Xuefu Zhuyu decoction.

| Plant name | Latin name | Chinese name | Medicinal  part | Ratio | Specimen number |
| --- | --- | --- | --- | --- | --- |
| *Prunus persica* (L.) Batsch | *Semen Persicae* | Tao Ren | Seed | 8 | 16080810 |
| *Carthamus tinctorius* L. | *Flos Carthami* | Hong Hua | Flower | 6 | 16121207 |
| *Angelica sinensis* (Oliv.) Diels | *Radix Angelicae Sinensis* | Dang Gui | Root | 6 | 16111801 |
| *Rehmannia glutinosa* (Gaertn.) DC. | *Radix Rehmanniae* | Sheng Di | Root | 6 | 16080402 |
| *Achyranthes bidentata* Blume. | *Radix Achyranthis Bidentatae* | Niu Xi | Root | 6 | 16121909 |
| *Paeonia lactiflora* Pall. | *Radix Paeoniae Rubra* | Chi Shao | Root | 4 | 16071607 |
| *Citrus × aurantium* L. | *Fructus Aurantii* | Zhi Qiao | Fruit | 4 | 16100905 |
| *Glycyrrhiza uralensis* Fisch. | *Radix Glycyrrhizae* | Gan Cao | Root | 4 | 16120303 |
| *Ligusticum striatum* DC. | *Rhizoma Chuanxiong* | Chuan Xiong | Root | 3 | 16120102 |
| *Platycodon grandiflorus* (Jacq.) A. DC. | *Radix Platycodonis* | Jie Geng | Root | 3 | 16102507 |
| *Bupleurum chinense* DC. | *Radix Bupleuri* | Chai Hu | Root | 2 | 16121903 |

The plant names have been checked with http://www.theplantlist.org.
