# Supplementary material for: Metabolomics Deciphers Potential Targets of Xuefu Zhuyu Decoction Against Traumatic Brain Injury in Rat
Source: Front Pharmacol. 2020 Sep 25;11:559618. doi: 10.3389/fphar.2020.559618 (PMC7546399; doi:10.3389/fphar.2020.559618)
Supplement: Supplementary file 2 [file Table_2.docx]

**Table 2**

Basic information on significant metabolites in different groups.

| Metabolites | Formula | KEGG | HMDB | CCI vs. sham  (3d) | | XFZYD vs. CCI  (3d) | | CCI vs. sham  (21d) | | XFZYD vs. CCI  (21d) | |
| --- | --- | --- | --- | --- | --- | --- | --- | --- | --- | --- | --- |
|  |  |  |  | VIP | FDR | VIP | FDR | VIP | FDR | VIP | FDR |
| Hypoxanthine | C_5_H_4_N_4_O | C00262 | 0000157 | 7.23 | 0.000 | 12.95 | 0.000 | 9.27 | 0.012 | 10.94 | 0.004 |
| Uridine | C_9_H_12_N_2_O_6_ | C00299 | 0000296 | 3.40 | 0.007 | 6.47 | 0.000 | 3.65 | 0.034 | 6.29 | 0.000 |
| L-Phenylalanine | C_9_H_11_NO_2_ | C00079 | 0000159 | 3.28 | 0.000 | 3.64 | 0.009 | 3.80 | 0.006 | 5.82 | 0.000 |
| L-Arginine | C_6_H_14_N_4_O_2_ | C00062 | / | 1.69 | 0.045 | 2.87 | 0.000 | 2.81 | 0.028 | 3.34 | 0.008 |
| Cinnamic acid | C_9_H_8_O_2_ | C10438 | 0000567 | 2.22 | 0.000 | 2.75 | 0.009 | 2.52 | 0.009 | 4.47 | 0.000 |
| L-Norleucine | C_6_H_13_NO_2_ | C01933 | 0001645 | 3.06 | 0.000 | 2.66 | 0.011 | 3.44 | 0.002 | 3.95 | 0.000 |
| L-Proline | C_5_H_9_NO_2_ | C00148 | 0000162 | 1.26 | 0.000 | 2.41 | 0.003 | 1.94 | 0.005 | 3.12 | 0.000 |
| L-Histidine | C_6_H_9_N_3_O_2_ | C00135 | 0000177 | 1.42 | 0.003 | 1.80 | 0.000 | 1.58 | 0.004 | 2.84 | 0.000 |
| L-Methionine | C_5_H_11_NO_2_S | C00073 | 0000696 | 1.02 | 0.007 | 1.63 | 0.000 | 2.21 | 0.000 | 1.86 | 0.000 |
| L-Valine | C_5_H_11_NO_2_ | C00183 | 0000883 | 1.28 | 0.000 | 1.18 | 0.024 | 1.86 | 0.000 | 1.21 | 0.039 |
| L-Tryptophan | C_11_H_12_N_2_O_2_ | C00078 | 0013609 | 1.11 | 0.000 | 1.57 | 0.000 | 1.55 | 0.011 | 1.94 | 0.000 |
| Choline | C_5_H_13_NO | C00114 | 0000097 | 5.72 | 0.000 | 7.18 | 0.000 | / | / | / | / |
| Gamma-Aminobutyric acid | C_4_H_9_NO_2_ | C00334 | 00112 | 4.76 | 0.000 | 5.03 | 0.000 | / | / | / | / |
| Dehydroascorb-ic acid | C_6_H_6_O_6_ | C00425 | 0001264 | 4.28 | 0.000 | 2.57 | 0.011 | / | / | / | / |
| 2-Pyrrolidinone | C_4_H_7_NO | C11118 | 0002039 | 1.83 | 0.000 | 1.86 | 0.000 | / | / | / | / |
| 4-Hydroxy-2-oxoglutaric acid | C_5_H_6_O_6_ | C01127 | 0002070 | 3.10 | 0.000 | 1.71 | 0.024 | / | / | / | / |
| Oleic acid | C_18_H_34_O_2_ | C00712 | 0000207 | 1.71 | 0.046 | 3.58 | 0.005 | / | / | / | / |
| 1,3,7-Trimethyluric acid | C_8_H_10_N_4_O_3_ | C16361 | 0002123 | / | / | / | / | 1.34 | 0.000 | 1.43 | 0.000 |
| 2-Hydroxycinna-mic acid | C_9_H_8_O_3_ | C01772 | 0002641 | / | / | / | / | 1.41 | 0.008 | 1.62 | 0.004 |
| alpha-D-Mannose 1-phosphate | C_6_H_13_O_9_P | C00636 | / | / | / | / | / | 2.63 | 0.000 | 1.98 | 0.004 |
